# Supplementary figures and images for: Temperature regulates synaptic subcellular specificity mediated by inhibitory glutamate signaling
Source: PLoS Genet. 2021 Jan 11;17(1):e1009295. doi: 10.1371/journal.pgen.1009295 (PMC7822552; doi:10.1371/journal.pgen.1009295)

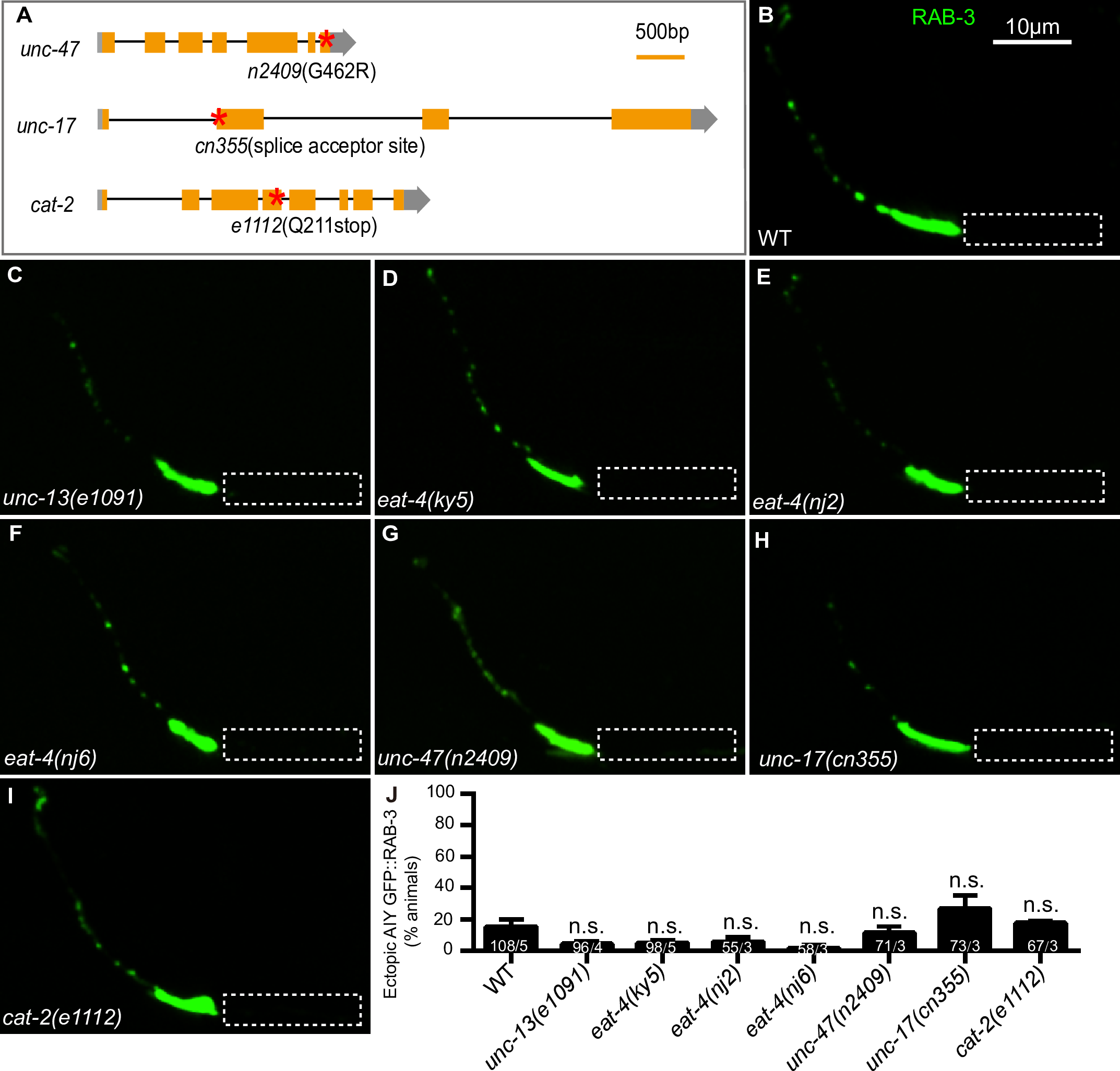

Supplement: S1 Fig — (A) Diagrams of the unc-47, unc-17 and cat-2 genomic structures, respectively. Exons and introns are indicated by boxes (yellow boxes are translated regions; gray boxes are untranslated regions) and black lines. Mutations are marked with asterisks. (B-I) Representative confocal micrographs of the AIY synaptic GFP::RAB-3 in wild-type (A), unc-13(e1091) (B), eat-4(ky5) (C), eat-4(nj2) (D), eat-4(nj6) (E), unc-47(n2409) (F), unc-17(cn355) (G) and cat-2(e1112) (H) animals at the adult Day 1 stage. Dashed boxes mark the zone 1 of AIY interneurons. The scale bar in (A) is 10μm and applies to (B-H). (J) Quantification of the percentage of animals with ectopic AIY synaptic marker GFP::RAB-3 in the zone 1 region for the indicated genotypes. The total number of independent animals (N) and the number of biological replicates (n) are indicated in each bar for each genotype (N/n). Statistics are based on one-way ANOVA with Dunnett’s test. Error bars are SEM. n.s., not significant. (TIF) [file pgen.1009295.s001.tif]

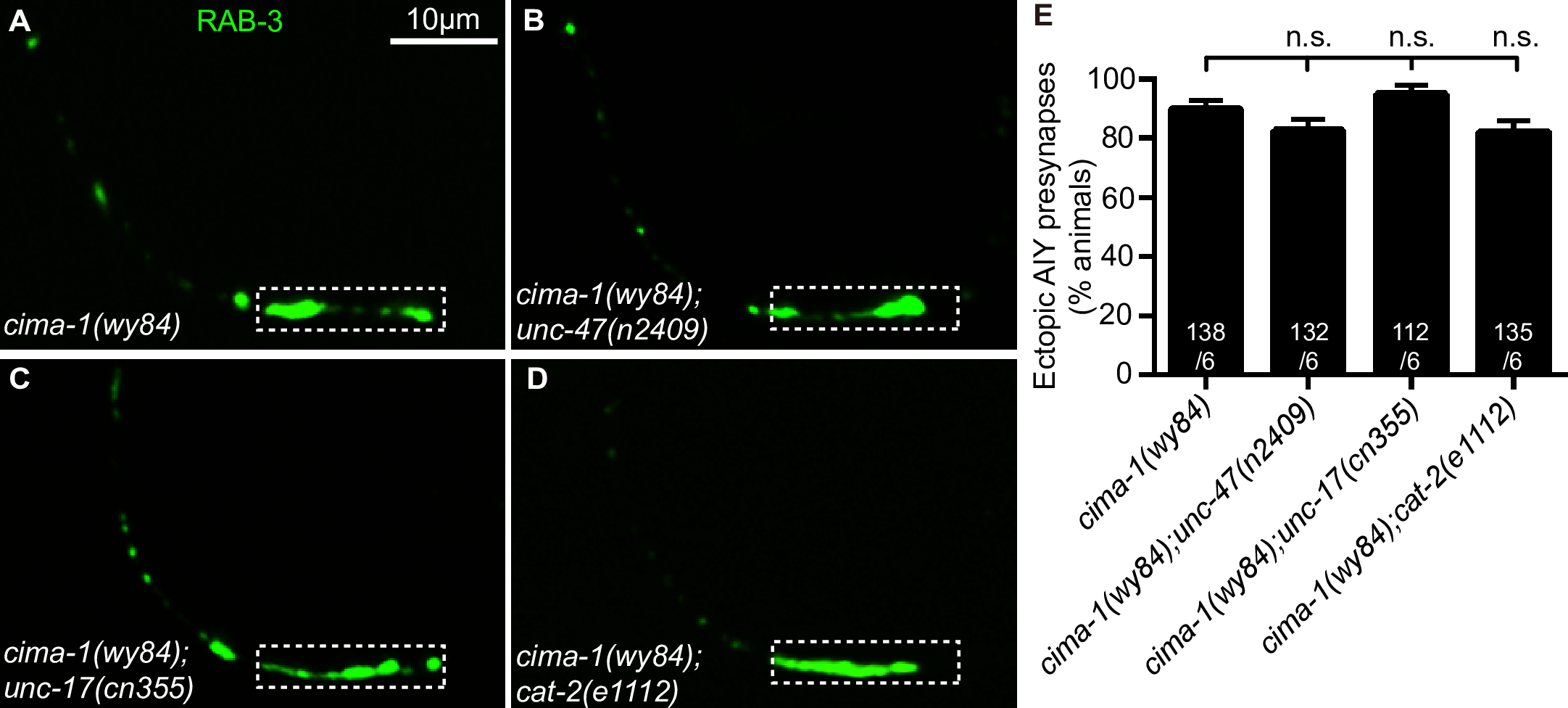

Supplement: S2 Fig — (A-D) Representative confocal micrographs of the AIY synaptic marker GFP::RAB-3 in cima-1(wy84) (B), cima-1(wy84);unc-47(n2409) (C), cima-1(wy84);unc-17(cn355) (D) and cima-1(wy84);cat-2(e1112) mutant (E) adult Day 1 animals. Dashed boxes mark the zone 1 region of AIY interneurons. The scale bar in (B) is 10μm and applies to (C-E). (E) Quantification of the percentage of animals with ectopic AIY synaptic marker GFP::RAB-3 in the zone 1 region. Note the ectopic synapses in cima-1(wy84) are not suppressed by mutations disrupting GABAergic (unc-47(n2409)), cholinergic (unc-17(cn355)) or dopaminergic (cat-2(e1112)) synaptic transmission. In the graph, the total number of independent animals (N) and the number of biological replicates (n) are indicated in each bar for each genotype as N/n. Statistics are based on one-way ANOVA with Dunnett’s test. Error bars are SEM. n.s., not significant. (TIF) [file pgen.1009295.s002.tif]

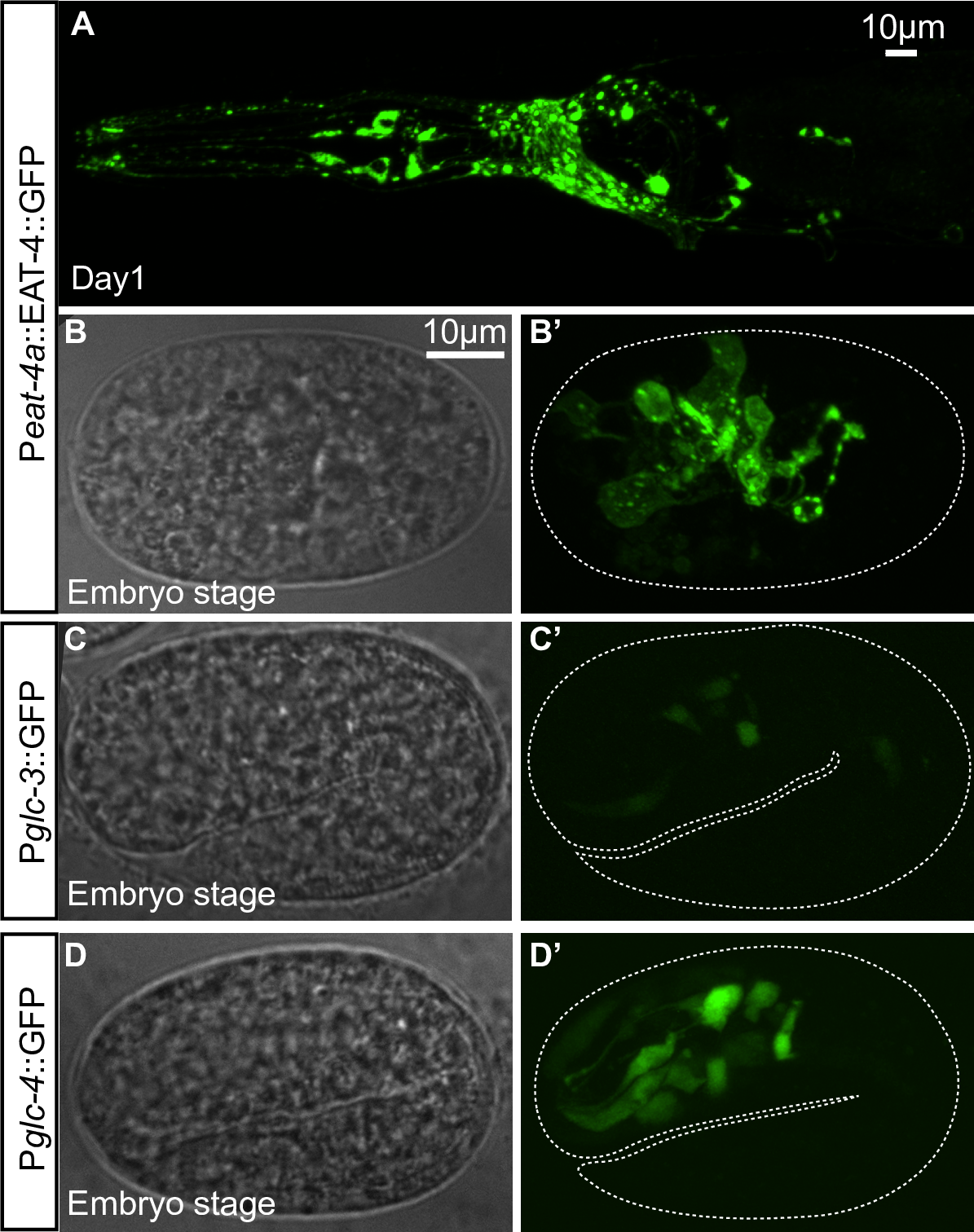

Supplement: S3 Fig — (A-B’) A representative confocal micrograph of eat-4 translational reporter (Peat-4a::eat-4::GFP). The expression of the reporter is enriched in the nervous system at the adult stage (A) and embryonic stage (B’). (B) is the corresponding bright field micrograph. (C and C’) A representative confocal micrograph of glc-3 transcriptional reporter (Pglc-3::GFP) at the embryonic stage (C’) and the corresponding bright field micrograph (C). (D and D’) A representative confocal micrograph of glc-4 transcriptional reporter (Pglc-4::GFP) at the embryonic stage(D’) and the corresponding bright field micrograph (D). The scale bars are 10μm, and the one in (B) applies to (B’, C, C’, D, D’). (TIF) [file pgen.1009295.s003.tif]

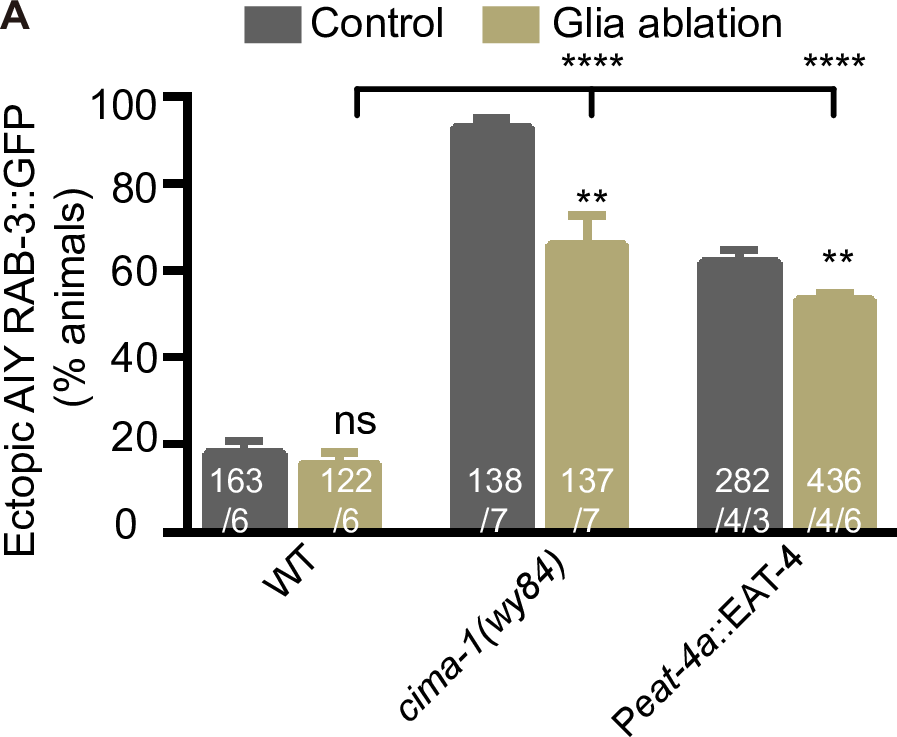

Supplement: S4 Fig — (A) Quantification of the percentage of animals with the ectopic AIY synaptic GFP::RAB-3 in the zone 1 region for the indicated genotypes. The data showed that VCSC glia only contribute partially to the synaptic subcellular specificity defect in either cima-1(wy84) or eat-4(OE) (Peat-4a::EAT-4) strains. Error bars are SEM. **P< 0.01, ****P< 0.0001, n.s., not significant. Statistics are based on one-way ANOVA with Dunnett’s test (the group of glia ablation) or unpaired t test (between the control group and the corresponding group of glia ablation). The total number of independent animals (N) and the number of biological replicates (n1) are indicated in each bar for each genotype, as are, for the transgenic lines created, the number of independent transgenic lines (n2) examined (using the convention N/n1 or N/n1/n2). (TIF) [file pgen.1009295.s004.tif]

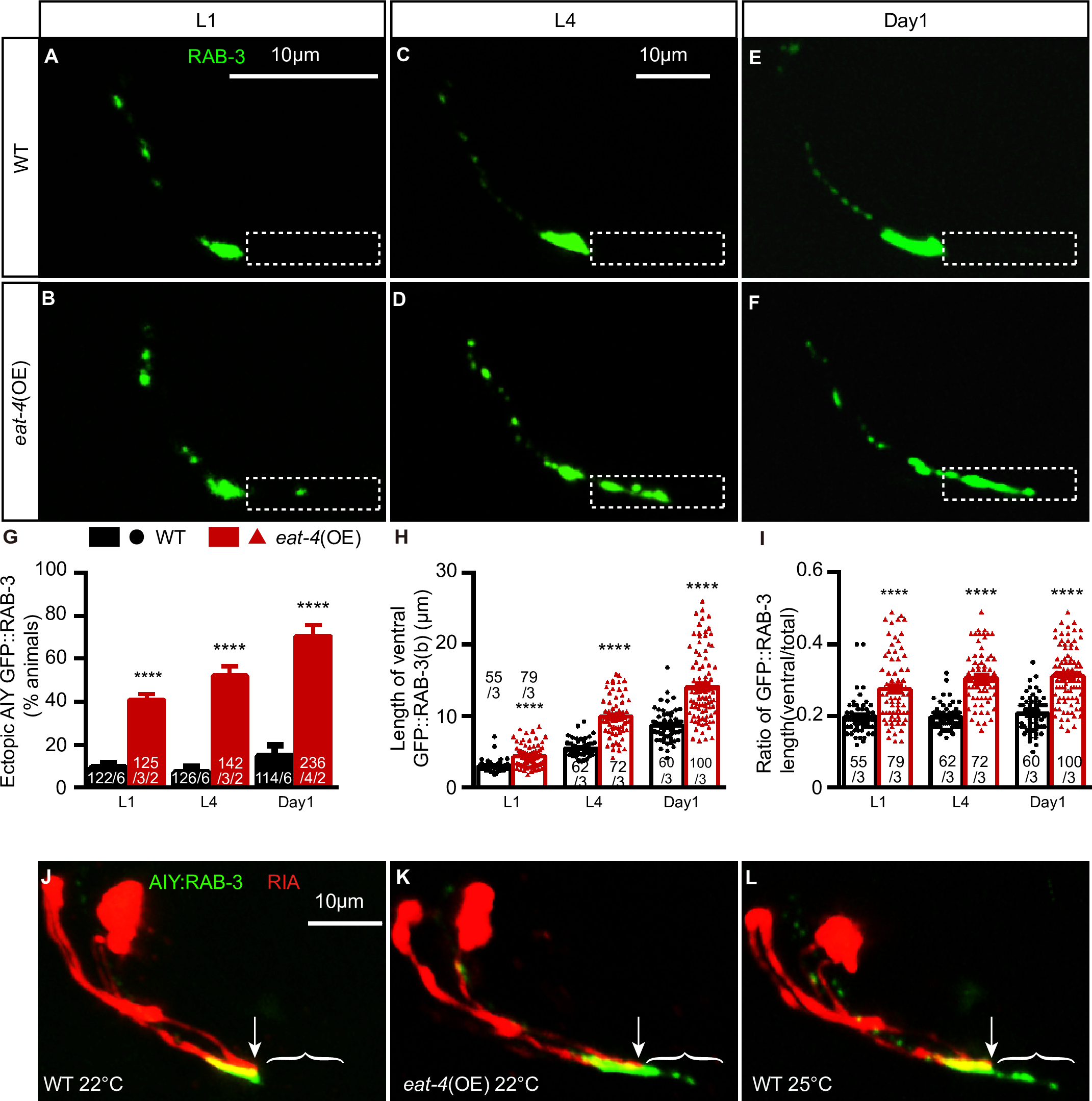

Supplement: S5 Fig — (A-F) Representative confocal micrographs of the AIY presynaptic marker GFP::RAB-3 in eat-4(OE) animals at different developmental stages. The presynaptic marker is not present in zone 1 region at larval L1 (A), L4 (C) or adult Day 1 stages (E) in wild type. However, the ectopic synapses appear in eat-4(OE) animals at larval L1 (B), L4 (D) and adult Day 1 stages (F), as indicated in the dashed boxes. Dashed boxes mark the zone 1 of AIY interneurons. The scale bars are 10μm, and the one in (A) applies to (B), in (C) applies to (D-F). (G-I) Quantification of the percentage of animals with the ectopic synapses in the AIY zone 1 (G), the ventral presynaptic length (H), and the ratio of the ventral to total presynaptic length (I) based on GFP::RAB-3. All quantification data consistently indicate that eat-4(OE) induces ectopic synapses since the newly hatched larval L1 stage. For (H) and (I), each spot represents the value from a single AIY. In the graph, the total number of independent AIY or animals (N) and the number of biological replicates (n1) are indicated in each bar for each genotype as N/n1. And for the transgenic lines created, the number of independent transgenic lines (n2) examined indicated in each bar for each genotype as N/n1/n2. For (H) and (I), one of transgenic lines in (G) was measured. Statistics are based on unpaired t test. Error bars are SEM. ****P< 0.0001. (J-L) Simultaneous visualization of GFP::RAB-3 in AIY and the postsynaptic RIA neurons (Pglr-3::mCherry) in wild-type animals cultivated at 22°C (J), 25°C (L) and eat-4(OE) animals (K). The arrows indicate the posterior endpoint of RIA. The AIY presynapses extend beyond the RIA endpoint in wild-type animals cultivated at 25°C (L) and eat-4(OE) animals (K). (TIF) [file pgen.1009295.s005.tif]

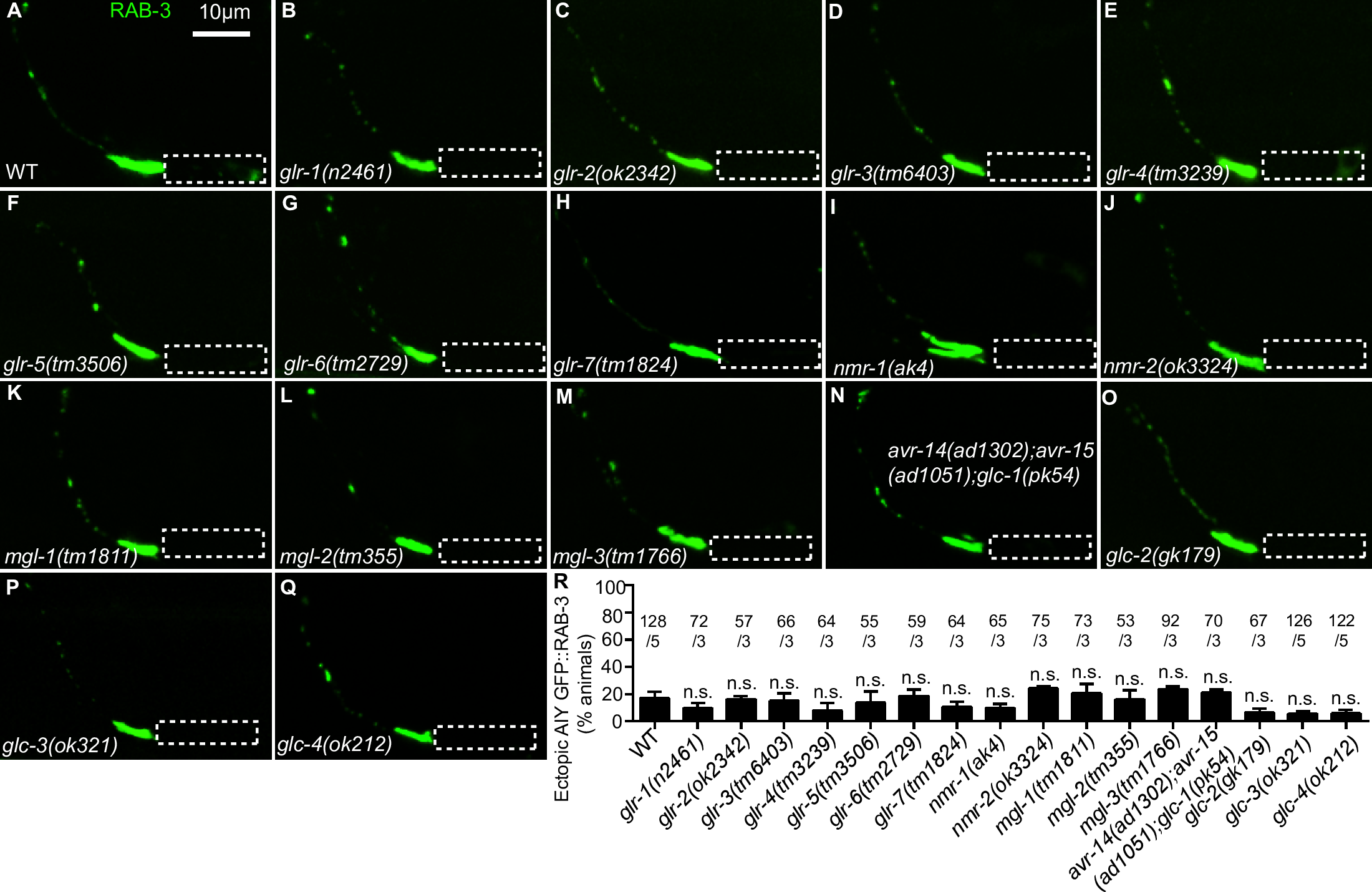

Supplement: S6 Fig — (A-Q) Representative confocal micrographs of AIY presynaptic marker GFP::RAB-3 in wild-type (A), glr-1(n2461) (B), glr-2(ok2342) (C), glr-3(tm6403) (D), glr-4(tm3239) (E), glr-5(tm3506) (F), glr-6(tm2729) (G), glr-7(tm1824) (H), nmr-1(ak4) (I), nmr-2(ok3324) (J), mgl-1(tm1811) (K), mgl-2(tm355) (L), mgl-3(tm1766) (M), avr-14(ad1302);avr-15(ad1501);glc-1(pk54) (N), glc-2(gk179) (O), glc-3(ok321) (P), glc-4(ok212) (Q) animals. In all images, dashed boxes correspond to zone 1 of AIY interneurons. The scale bar in (A) is 10μm, applying to (B-Q). (R) Quantification of the percentage of animals with the ectopic AIY synaptic marker GFP::RAB-3 corresponding to (A-Q). The data show that none of those glutamate receptors is required for synaptic subcellular specificity per se. The total number of independent animals (N) and the number of biological replicates (n) are indicated in each bar for each genotype (N/n). Statistics are based on one-way ANOVA with Dunnett’s test. Error bars are SEM. n.s., not significant. (TIF) [file pgen.1009295.s006.tif]

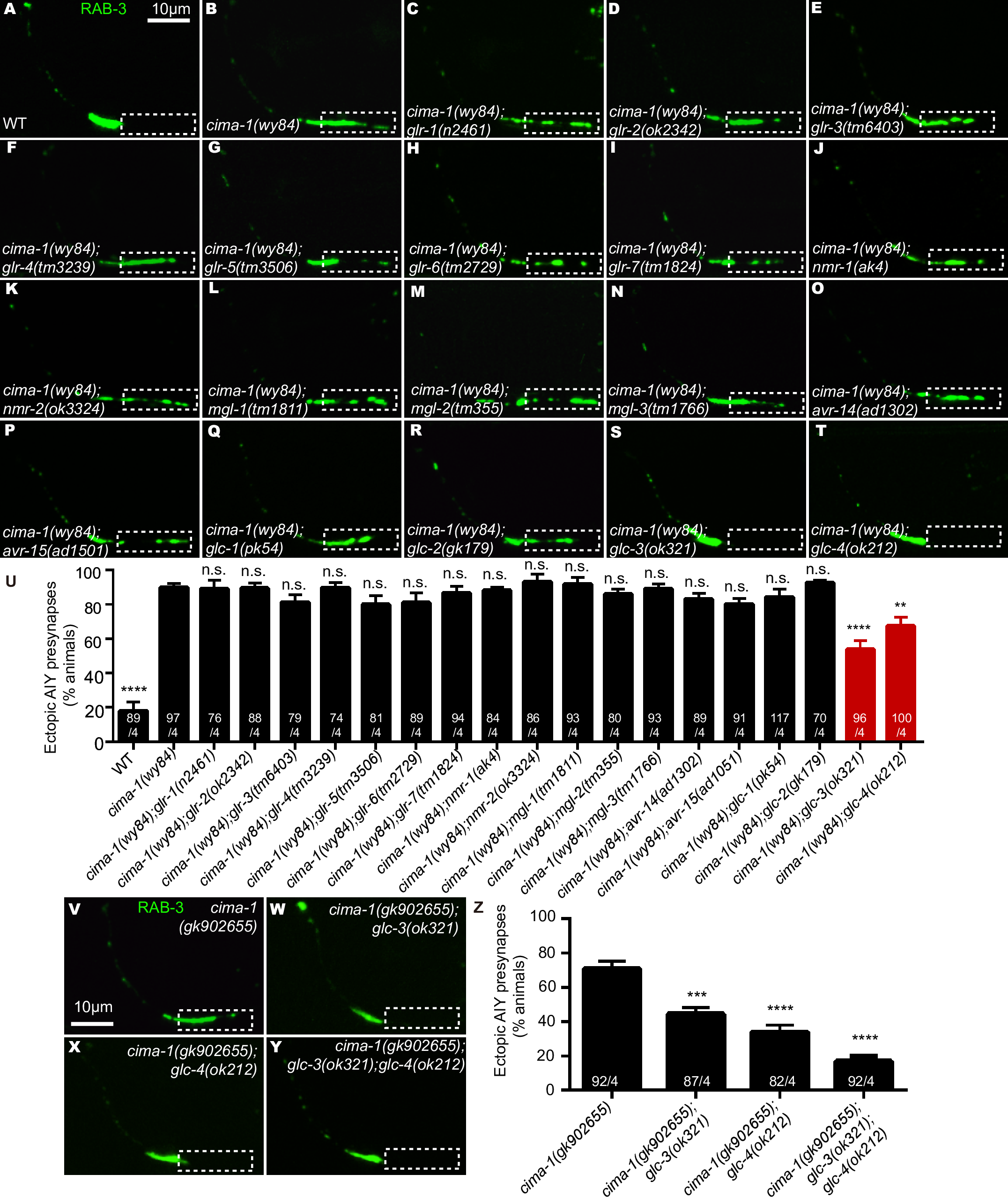

Supplement: S7 Fig — (A-T) Representative confocal micrographs of AIY presynaptic marker GFP::RAB-3 in wild type (A), cima-1(wy84) (B), cima-1(wy84); glr-1(n2461) (C), cima-1(wy84);glr-2(ok2342) (D), cima-1(wy84);glr-3(tm6403) (E), cima-1(wy84); glr-4(tm3239) (F), cima-1(wy84); glr-5(tm3506) (G), cima-1(wy84); glr-6(tm2729) (H), cima-1(wy84); glr-7(tm1824) (I), cima-1(wy84); nmr-1(ak4) (J), cima-1(wy84);nmr-2(ok3324) (K), cima-1(wy84);mgl-1(tm1811) (L), cima-1(wy84);mgl-2(tm355) (M), cima-1(wy84);mgl-3(tm1766) (N),cima-1(wy84); avr-14(ad1302) (O), cima-1(wy84);avr-15(ad1501) (P), cima-1(wy84);glc-1(pk54) (Q), cima-1(wy84);glc-2(gk179) (R), cima-1(wy84);glc-3(ok321) (S), cima-1(wy84);glc-4(ok212) (T). GLC-3 and GLC-4 partially mediate the ectopic presynaptic specificity in cima-1(wy84). In all images, dashed boxes correspond to zone 1 of AIY interneurons. The scale bar in (A) is 10μm, applying to (B-T). (U) Quantification of the percentage of animals with ectopic AIY synaptic marker GFP::RAB-3 in the zone 1 region corresponding to (A-T). (V-Y) Representative confocal micrographs of AIY presynaptic marker GFP::RAB-3 in cima-1(gk902655) (V), cima-1(wy84);glc-3(ok321) (W), cima-1(wy84);glc-4(ok212) (X), cima-1(wy84);glc-3(ok321);glc-4(ok212) (Y) mutants. The dashed boxes correspond to zone 1 of AIY interneurons. The scale bar in (V) is 10μm, applying to (W-Y). (Z) Quantification of the percentage of animals with the ectopic AIY synaptic marker GFP::RAB-3 in the zone 1 region corresponding to (V-Y). For U and Z, the total number of independent animals (N) and the number of biological replicates (n) are indicated in each bar for each genotype (N/n). Statistics were based on one-way ANOVA with Dunnett’s test. Error bars are SEM. **P< 0.01, ***P< 0.001, ****P< 0.0001, n.s., not significant. (TIF) [file pgen.1009295.s007.tif]

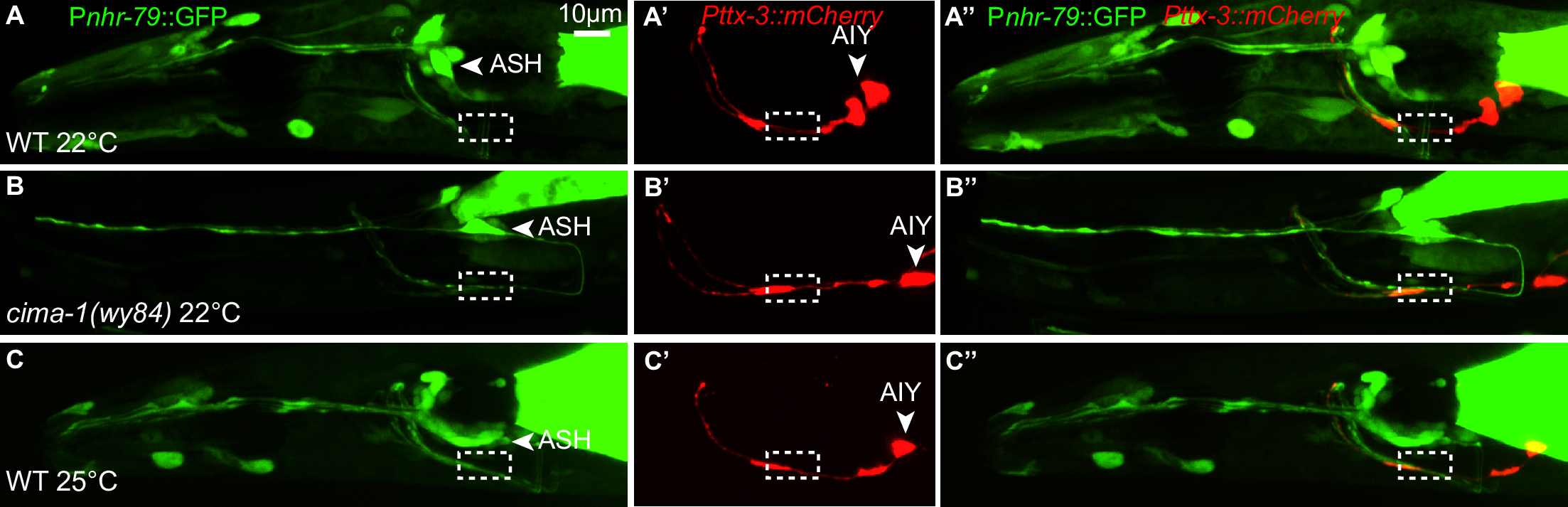

Supplement: S8 Fig — (A-C”) Representative confocal micrographs of ASH (Pnhr-79::GFP) (A, B, C) and AIY cytoplasmic marker (Pttx-3::mCherry) (A’, B’, C’) at the adult Day 1 of wild-type animals cultivated in 22°C (A, A’), 25°C (C, C’) and cima-1(wy84) (B, B’) animals. A”, B” and C” are the corresponding merged channels. We noticed that the ASH axons extend posteriorly overlapping with AIY in zone 1 in cima-1(wy84) or wild-type animals cultivated in 25°C animals. The dashed boxes correspond to zone 1 of AIY interneurons; the white arrow heads mark the ASH or AIY soma; the scale bar in (A) is 10μm and applies to the A’-C”. (TIF) [file pgen.1009295.s008.tif]

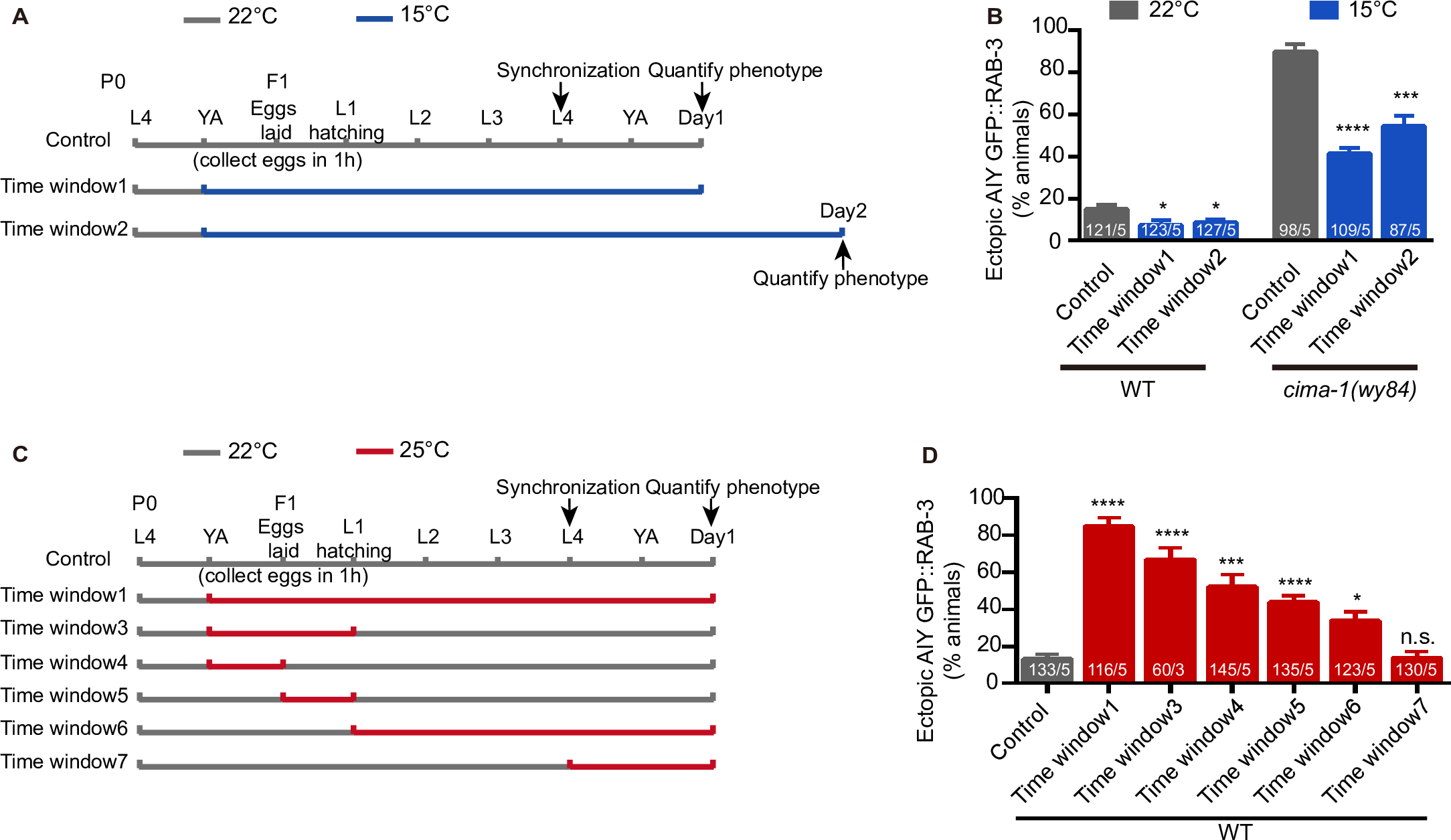

Supplement: S9 Fig — (A) A schematic diagram shows the low cultivation temperature conditions. The control group was cultivated at the constant 22°C condition (gray line). The low temperature group was transferred from 22°C (gray line) into 15°C (blue line) since the parent generation (P0) young adult stage until the next generation (F1) adult Day 1 or Day 2 stage when the phenotype was scored. (B) Quantification of the percentage of animals with ectopic AIY synapses in the zone 1 region at 15°C for wild-type and cima-1(wy84) mutants. Animals grown at 15°C show significant less ectopic synapses than at 22°C for both wild-type and cima-1(wy84). (C) A schematic diagram shows the high cultivation temperature conditions (25°C, red line) in different time windows. (D) Quantification of the percentage of animals with the ectopic AIY synaptic marker GFP::RAB-3 in the zone 1 region. Noted that both embryonic and larval stages are sensitive to the high temperature, the embryonic stage is more sensitive (compare window 3 and window 6). No ectopic synapses were observed when animals were treated after L4 stage (window 7). For (B) and (D), the total number of independent animals (N) and the number of biological replicates (n) are indicated in each bar for each genotype (N/n). Statistics are based on one-way ANOVA with Dunnett’s test. Error bars are SEM. *P< 0.05, ****P< 0.0001, n.s., not significant. (TIF) [file pgen.1009295.s009.tif]

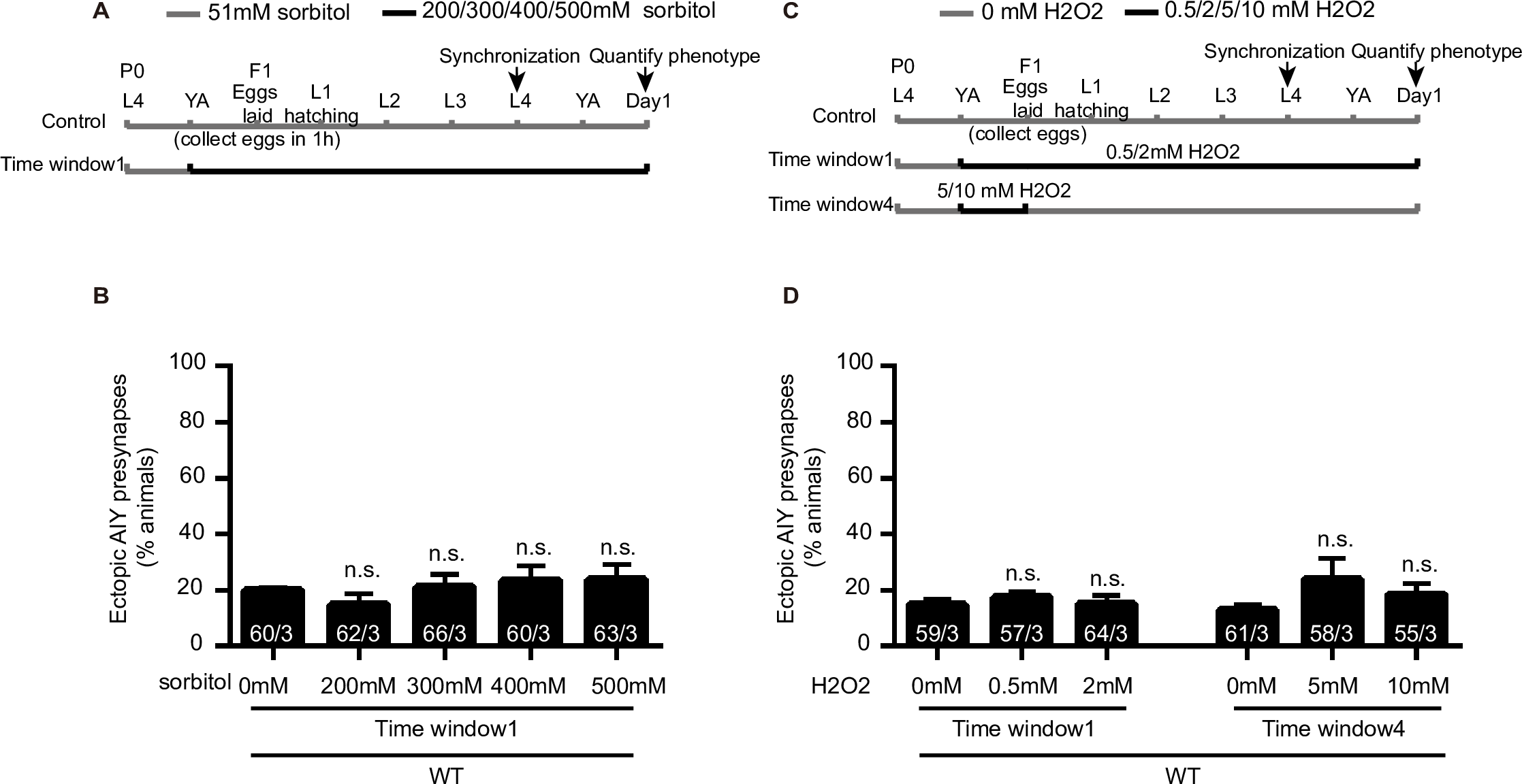

Supplement: S10 Fig — (A) A schematic diagram shows the time window for the sorbitol treatment. Young adults were grown on NGM agar plates containing 0mM (control, gray line), 200mM, 300mM, 400mM or 500 mM sorbitol (black line) seeded with OP50 until the next generation (F1) adult Day 1 when the phenotype was scored. (B) Quantification of the percentage of animals with ectopic AIY synapses in the zone 1 region under different concentration of sorbitol. The data show that the osmotic stress with the concentration of 500mM or less sorbitol has no effect on the AIY synaptic subcellular specificity. (C) A schematic diagram shows time window for the oxidative stress treatment. Young adults were grown on NGM agar plates with OP50 with 0mM (control, gray line), 0.5mM, 2mM, 5mM or 10mM hydrogen peroxide (black line) in the specified time window. The phenotype of the next generation (F1) was scored at the adult Day 1 stage. (D) Quantification of the percentage of animals with the ectopic AIY synaptic marker GFP::RAB-3 in the zone 1 region corresponding to (C). The data show that the oxidative stress conditions do not affect the AIY synaptic subcellular specificity. For (B) and (D), the total number of independent animals (N) and the number of biological replicates (n) are indicated in each bar for each genotype (N/n). Statistics are based on one-way ANOVA with Dunnett’s test. Error bars are SEM. n.s., not significant. (TIF) [file pgen.1009295.s010.tif]
