# Supplementary material for: Temperature regulates synaptic subcellular specificity mediated by inhibitory glutamate signaling
Source: PLoS Genet. 2021 Jan 11;17(1):e1009295. doi: 10.1371/journal.pgen.1009295 (PMC7822552; doi:10.1371/journal.pgen.1009295)
Supplement: Attachment — Submitted filename: Response to reviewers.pdf [file pgen.1009295.s016.pdf]

Zhiyong Shao
Professor of Institutes of Brain Science Fudan University
138 Yixueyuan Rd, Mingdao Bldg 1016, Shanghai
Shanghai, 200032, China
Phone: +86-21-54237662
Email: shaozy@fudan.edu.cn

Dear Dr. Anne Hart and reviewers,

Enclosed please find our manuscript now entitled "Temperature regulates synaptic subcellular specificity mediated by inhibitory glutamate signaling". We received the constructive comments from the reviewers. We integrated all their suggestions, including analyzing new alleles, neuronal activities, and editing the text with more background information etc. We believe that the revised MS is improved significantly to better underscore its main findings.

Briefly, we showed that high temperature (25°C) promotes, and low temperature (15°C) suppresses the ectopic synaptic formation, while other stresses have no effect; we further confirmed the role of ASH in promoting the AIY synaptic specificity deficits by ASH-specific expressing *eat-4* or cell ablation; we showed that ASH specific *eat-4(OE)* inhibits AIY activity through calcium imaging; we took out the GRASP data due to the unexpected signaling in zone 3 region, and changed the EM images to better illustrate the ASH-AIY synaptic connection; we also discussed more literatures on the role of neural activity in development and the connection between AIY and temperature. Please find the point-to-point responses below.

I very much thank you for your thoughtful comments. I hope you agree that integration of all their comments resulted in a stronger and significant

manuscript that would benefit the readership of *PLOS genetics*.

Thank you for reconsidering our manuscript.

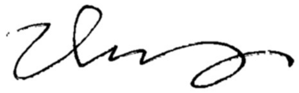

Professor of Institutes of Brain Science
Fudan University
Tel: +86-21-54237762
Email: shaozy@fudan.edu.cn

Point-by-point response to the reviewers' comments

Editor:

1) address if high temperature is uniquely able to drive ectopic synapse formation (versus other stressors)

Answer:

This is an excellent point. Actually, we don't think the phenotype is simply due to the high temperature stress for three reasons. First, we also observed the suppression of the ectopic synapses by low cultivation temperature. Therefore, this synaptic phenotype is not only affected by high temperature, but also by low temperature. Secondly, we also examined high osmotic stress and oxidative stress, and found neither of them affects the AIY presynaptic distribution. Thirdly, the 25°C induced phenotype is *eat-4/glc-3/glc-4*-dependent, and specifically require *eat-4* in ASH. Therefore, we think the ectopic phenotype induced by 25°C is specifically through activating ASH neurons, but not through a general stress signaling pathway. Those data were added to the revised version **(Fig 7I-7R and S9B, S10 Fig).**

2) provide clarity regarding the data/results. A data table that gives results for all studies, including breaking out the individual transgenic lines reported and the p-values/statistical score, must be provided or accessible at a permanent location listed in the manuscript. For example, extrachromosomal lines were presumably scored separately and they should be reported separately in the supplementary materials, although it is certainly appropriate to merge these in the main text figures, if the lines have essentially the same impact on phenotype for each assay.

Answer:

In revised version, we have provided the archive of raw data for figures in **S3 Excel**. Thanks for the suggestion.

3) explicitly state which studies were undertaken by researchers blinded as to genotype/treatment.

Answer:

Thanks for asking. The ectopic synaptic phenotype is quantified blindly (Figs 1, 2, 3, 4,7, and S1, 2, 4, 5, 6, 7, 9, 10 Figs). Other data are quantified based on the genotype/treatment, which is now stated in the method section.

4) use the acknowledgement required by the CGC, with wording recommended at their website.

Answer:

We did this. Thanks for reminding.

5) corrects typos/errors in nomenclature. For example, C. elegans gene names should not be capitalized, even at the beginning of a sentence.

Answer:

We corrected those. Thank you.

Reviewer #1:

Review of "Temperature regulates synaptic subcellular specificity through glutamatergic signaling". This is an interesting manuscript examining the role of activity in synaptic development. The entre to the work was the identification of mutations in cima-1, a protein that appears to be a sialic acid transporter, in a screen for AIY synaptic defects. The loss of cima-1 leads to AIY neurons forming ectopic synapses in a region where they normally do not make synapses. Here the authors link that defect to glutamatergic signaling via 2 glutamate-gated chloride channels. The work is rigorous, the results novel and conclusions justified. That manipulating inhibits or promotes ectopic (or inappropriate) synapse formation is very exciting. There is an impressive amount of work that has been done very carefully and thoroughly, and will be of interest to the general field.

All of that being said, 1) I felt there was a relative lack of consideration of the broader literature. There ares several papers examining the role of calcium channels, neurotransmitters and kinsesin-dependent transport regulation of synaptic development, none of which is referenced. One example, but not the only one, would be the 2003 Gally and Bessereau paper documenting that unc-25 mutants exhibit normal synaptogenesis, or the unc-104 mutants documenting synapses will form at cell bodies in some transport mutants. Glutamate released from glia regulate extrasynaptic glutamate receptor accumulation in many contexts, first identified in Drosophila, and is now documented in many systems. There is an interaction between glial glutamate and dopamergic neuron survival in C. elegans. These papers do nothing to diminish the novelty of the work presented here, but provide a framework for why activity, per se, is not required for normal synapse formation. Thus, my only major concern is the lack of attention to the previous literature in the field.

Answer:

We thank the reviewer for the positive comment. Now we add a paragraph **(second paragraph)** in the introduction specifically discussing the role of neuronal activity in neuronal development, including those finding in *C. elegans* and flies. And discussion about the role of glutamate in neuronal physiology and pathology is added in the discussion **(lines 491-495)**.

2) I have an only a few minor things I think should be addressed before being acceptable for publication. Line 24 - "induced severely ectopic synapses" could the authors describe what makes the ectopic synapses "severe" I'm not sure I understand what that means.

Answer:

We thank the reviewer for pointing this out. "Severely" here means "robustly". Now we replaced with "robustly".

3) Given the ~20% of wild-type animals with ectopic synapses, could the authors comment whether growth at 15 eliminates this?

Answer:

Excellent idea. Thank you.

In the revision, we grew the wild-type and *cima-1(wy84)* at 15°C and found that low temperature indeed suppresses the ectopic synapses for both genotypes **(S9B Fig)**.

4) Does the structure of the AIY neuron (outgrowth, branching, etc.) change appreciably when animals are reared at 25°C?

Answer:

The reviewer is asking if the high temperature affect the global AIY morphology. In the revision, we labeled the AIY process with cytoplasmic mCherry and found that it slightly extended posteriorly, but no other obvious changes at 25°C **(S8A', S8C' Fig).** Thank you.

5)In the eat-5 mutants do the GLC-3 or GLC-4 proteins accumulate in zone 1?

Answer:

The reviewer is asking if GLC-3 and GLC-4 protein localized to the AIY zone 1 region in the *eat-4* mutants. No, we did not observe the accumulation of GLC-3 or GLC-4 in the zone 1 in *eat-4* mutants, we added those data in the revision **(Fig 5B, 5B', 5G, 5G')**.

Reviewer #2:

Neuronal activity can affect synapse formation, but the underlying molecule mechanisms are still unclear. In this manuscript, the authors use the C. elegans AIY neurons as a model to address this question. Although the observations are interesting, the main conclusions are not well supported by the data, and many results were misinterpreted.

Major issues

1) The authors started from analyzing cima-1 suppressors and showed that unc-13(lf), eat-4(lf), glc-3(lf), and glc-4(lf) suppressed cima-1 (wy84) phenotype. As wy84 is a missense mutation, the suppression of wy84 could be allele specific, and it is necessary to confirm their phenotypes using a deletion allele of cima-1.

Answer:

Thank the reviewer for the comments. Since no *cima-1* deletion is available, we tested another *cima-1* allele *gk902655*, a nonsense mutation of *cima-1*(R476 to opal stop codon) which phenocopies *cima-1(wy84)*[1, 2]. We confirmed the role of *unc-13(e1091)*, *eat-4(ky5)*, *glc-3(ok321)*, *glc-4(ok212)* in suppressing *cima-1* using *gk902655* **(Fig 1I-1K, 1O, S7V-S7Z Fig).** Those data suggest that the suppression of *cima-1* by *unc-13*, *eat-4*, *glc-3* and *glc-4* is not allele-specific.

2) Based on the suppression of cima(wy84) by eat-4(lf) and the inducement of the cima(wy84)-

like phenotypes by overexpressing eat-4, the author concluded that the release of Glutamate was required for AIY synapse formation. eat-4 is essential for transport of Glu into synaptic vesicles, but eat-4(lf) also displays other notable phenotypes, such as eating disorder, defects in sensing temperature change, which may or may not associated with Glu releases from synapse terminals. To show that the cause of suppression of cima(wy84) by eat-4 is due to Glu release, the author can supply cima(wy84);eat-4 animal with Glu to examine the release of suppression, and the detail method of this treatment can be found in Leon Avery 1997 EMBO J paper (or other Avery lab's manuscripts). The author also claimed that eat-4(over expression) caused "Glutamatergic neuon overactivation" and "over release of Glutamate", which were not based on any data. EAT-4 transports Glu into synaptic vesicles, which is unlikely to activate "Glutamatergic neuon". As the release of Glu-contained synaptic vesicles depend on neuronal activity, overexpression of eat-4 is also unlikely enough to cause ove-release of Glu. By saying that, even if overexpression of eat-4 could overload Glu into synaptic vesicles, those glutamate may not be able to release appropriately without stimulation of ASH neurons. To support the conclusion, the author need to show overexpression of eat-4 can active ASH neurons by calcium imaging, and the increase release of Glu using VGLUT-pHluorin (see Bargmann lab 2017 Elife paper).The Glutamatergic neuronal identity of ASH is regulated by unc-42 in a very specific manner (see Hobert lab 2013 Cell paper), and the author may want to confirm the function ASH neurons by examining the role of unc-42. The function of ASH neurons can also be confirmed by killing ASH neurons using laser ablation or ced-3 overexpression. It was motioned in the manuscript that the activation of GLC-3 GLC-4 by Glu could suppress the activity of AIY neurons and cause the phenotypes, but no evidence was shown. It could be tested by silencing AIY neurons by expression of gain of function potassium channels (such as, slo-19(gf), UNC-103(gf), see Bargmann lab 2016 Cell paper).

Answer:

Thank the reviewer for all great suggestions.

First, we agree that *eat-4*(over expression) is not necessary to cause "Glutamatergic neuron overactivation". We corrected the wording in the revision. Thanks for the reminding.

Here we believe that the reviewer was asking the following four major questions: 1) Does *eat-4* function through glutamatergic neurons? 2) Could *eat-4(OE)* results in glutamate over-release? 3) Is the glutamate from ASH? 4) Can GLC-3/GLC-4 activated by glutamate suppress AIY activity and cause the synaptic distribution defect?

1) So far, we know that *eat-4* is only expressed in glutamatergic neurons and the only known EAT-4 function is pumping glutamate into synaptic vesicles [3-5]. The defects in *eat-4(lf)* mutants are therefore most likely due to the glutamate release defect. In our studies, through tissue-specific rescue, we also found that *eat-4* acts mainly in ASH glutamatergic neurons **(Fig 2B)**, which further supports the hypothesis that *eat-4* regulates the AIY synaptic subcellular specificity through glutamatergic signaling.

2) VGLUT overexpression leads to increasing glutamate loading in the synaptic vesicle and enhancing glutamate release in *Drosophila* [6, 7] and vertebrates [8, 9].

To test this possibility in *C. elegans*, we examined the spontaneous AIY activity by AIY-specific GCamP6s. We observed spontaneous Ca2+ oscillation in AIY in wild type. Remarkably, the frequency of Ca2+ oscillation in AIY is significantly reduced in ASH-specific *eat-4(OE)* worms **(Fig 4I-4K, S1 Video)**. Those data strongly support the model that ASH-specific *eat-4(OE)* affects the AIY activity by over releasing glutamate.

3) Firstly, our tissue-specific rescue data suggest that ASH is the major action site for *eat-4* **(Fig 2A-2B and 7R)**; secondly, the EM data also showed that the ASHs are AIY presynaptic neurons **(Fig 6);** thirdly, as suggested by the reviewer, we performed ASH ablation and found that the ablation indeed suppresses the ectopic synaptic structure in both *cima-1(wy84)* and *eat-4(OE)* animals **(Fig 2D)**; fourthly, we did ASH EAT-4::PHluorin imaging for animals cultivated at 22°C and 25°C and found that the high temperature enhances the PHluorin intensity, suggesting that ASH is more active at higher temperature **(Fig 7S-T, 7U)**. Collectively those data indicate that ASHs are the major glutamate neurons that regulate AIY synaptic specificity.

4). Our genetic data support a model that glutamate released from ASH activates GLC-3/GLC-4 in AIY to silence AIY and promote the ectopic synaptic formation. Five lines of evidence support this. First, the loss of function mutations of *eat-4*, *glc-3/glc-4* suppresses the ectopic synapse **(Fig 1O, 3F)**; secondly, the tissue specific rescue data indicates *eat-4* and *glc-3/glc-4* act in ASH and AIY respectively **(Fig 2B, 4C)**; thirdly, overexpressing *eat-4* in ASH or *glc-3/glc-4* in AIY results in the ectopic synaptic formation **(Fig 2C, 4H)**; fourthly, as suggested by the reviewer, we expressed the *unc-103(gf)* in the AIY and indeed found that the transgene induces the ectopic synapse just as *eat-4* or *glc-3/glc-4* overexpression **(Fig 4G-H).** Fifthly, the frequency of the AIY spontaneous Ca^{2+} oscillation is significantly reduced by ASH-specific *eat-4(OE)* **(Fig 4I-4K, S1 Video)**. Altogether, those data strongly support our model that glutamate released from ASH activates GLC-3/GLC-4 in AIY to silence AIY and promote the ectopic synaptic formation.

<u>3) The link between environmental temperature and synaptic phenotypes is weak. Based on the suppression of "extra" synapses by eat-4 in 25C, the author concluded that the</u>

“glutamatergic activity is required for the high temperature”, again without direct evidence. To reach the conclusion, one needs to show 25C can activate ASH and increase Glu release from ASH. eat-4 has been shown to play a role in sensing temperature changes in worms, and it is possible the suppression caused by eat-4 is due to insensitive to temperature.

Answer:

We agree that in the previous version, the link between high temperature and glutamate release is indirect. In the revision, we examined the ASH neuronal activity by imaging EAT-4::PHluorin and found the PHluorin intensity is higher at 25°C than at 22°C **(Fig 7S-7T, 7U)**. Additionally, we showed that the AIY ectopic synaptic formation require ASH-specific *eat-4* **(Fig 7R).** Those data further support the model that high temperature promotes glutamate release from ASH.

4) Based on data from eat-4 rescue and EM study, the author concluded that Glu released from ASH could activate GLC-3 GLC-4 in AIY neurons to regulate synapse formation. However, GLC-3 and GLC-4 are both concentrated in the AIY presynaptic buttons (to the downstream neurons), not at the post-synaptic region of ASH-AIY synapses. The question is how the Glu released from ASH neurons to activate GLC-3/GLC-4 residing outside of ASH-AIY synapses. One possibility is the “over released” Glu may diffuse to reach GLC-3/GLC-4, but this will be inconsistent with the eat-4 rescue data, in which expression of eat-4 in other glutamatergic neurons did not rescue the phenotype. Some explanation or model will be needed to facility the understanding of the data.

Answer:

Here the reviewer is asking how the glutamate released from ASH reaches the GLC-3/GLC-4 receptor on AIY since both receptors localize on the presynaptic site; and if the glutamate diffuses to GLC-3/GLC-4, why other glutamate neurons do not regulate AIY synaptic specificity. Those are all excellent questions.

GLC-3 and GLC-4 could localize to both presynaptic and postsynaptic sites since they are closely aligned in the same region in AIY [10]. Glutamate from ASH could directly activate the GLC-3/GLC-4 at the ASH-AIY synaptic sites. Alternatively, it may activate the presynaptic GLC-3/GLC-4 receptors.

Regarding why other glutamate neurons do not affect AIY presynaptic subcellular specificity, there are three possible reasons. First, the total amount glutamate released is different. Second, the timing of glutamate release is different. Thirdly, the site of the glutamate release is different. We now discussed those possibilities in the discussion **(lines 460-469)**.

Reviewer #3:

In this manuscript, Wang et al. show that the C. elegans interneuron AIY forms ectopic

presynapses in specific genetic backgrounds or after cultivation of worms at high temperature. Wang et al. show that these ectopic presynapses are regulated by the vesicular glutamate transporter EAT-4 in ASH and postsynaptic glutamate-gated chloride channels GLC-3 and GLC-4 in AIY. The authors previously showed that presynaptic markers (RAB-3 and SYD-1) accumulate in an asynaptic region of AIY (defined as zone 1) in mutants lacking the sialin homolog CIMA-1 in a manner that is partly dependent on the VCSC glia. This study investigates whether neurons contribute to the formation of these ectopic presynapses. Wang et al. show that the ectopic RAB-3 puncta observed in cima-1 mutants are suppressed by mutants lacking the synaptic vesicle priming protein unc-13 or the vesicular glutamate transporter eat-4. In an impressive set of rescue experiments, they use a large panel of cell-specific promoters to reveal that eat-4 acts largely in the glutamatergic neuron ASH to regulate these ectopic synapses. Overexpression (OE) of eat-4 under its own promoter or promoters driving expression in ASH also result in ectopic RAB-3 puncta. A thorough analysis of potential glutamate receptors identified the glutamate-gated chloride channels GLC-3 and GLC-4 as being required for ectopic presynapse formation. Rescue experiments show that glc-3 and glc-4 function in AIY, and in a nice experiment the authors show that overexpression of glc-3 and glc-4 in AIY induces ectopic RAB-3 puncta that are dependent on eat-4, and thus likely dependent on presynaptic glutamate. Subcellular localization studies show that mCherry-tagged GLC-3 and GLC-4 appear to localize to presynaptic areas in AIY and mislocalize to zone 1 in cima-1 mutants and in eat-4(OE) animals. Wang et al. use EM and GRASP to identify previously undescribed contacts between ASH and AIY (although see comments below), providing a direct explanation for how eat-4 in ASH could regulate GLC-3 and GLC-4 in AIY. Lastly, the authors show that cultivating worms at high temperature (25 degrees C) surprisingly results in ectopic RAB-3 puncta and this effect is dependent on eat-4 and glc-3 and glc-4. The experiments are thorough and rigorous and the data are, for the most part, convincing. There are several novel findings in this manuscript including the observation that glutamate regulates the formation of ectopic presynapses in AIY via chloride-gated channels, that the glutamatergic ASH neuron potentially makes synapses with AIY, and that high cultivation temperature induces ectopic presynapses.

Major comments

1) Do the ectopic RAB-3 and SYD-1 puncta in zone 1 represent true ectopic synapses or just ectopic accumulation of presynapses without corresponding postsynapses? Either evidence from prior publications or from experiments should be provided, or statements referring to "ectopic synapses" should be toned down.

Answer:

The reviewer is asking whether the AIY presynaptic (RAB-3 or SYD-1) structure has a corresponding postsynaptic structure.

Thank the reviewer for the excellent question. Unfortunately, we don't have the answer for now. In *cima-1(wy84)* mutants, one of AIY postsynaptic neuron RIA also partially extends posteriorly, but not overlapping with the ectopic synapses. And, in the EM data, we found neurons of unknown identity appose to the AIY ectopic presynaptic sites [1]. Unlike the postsynaptic structure in vertebrates, *C. elegans* postsynaptic site does not have postsynaptic density.

Therefore, we cannot conclusively determine if the neuron aligned to the presynaptic site is a real AIY postsynaptic neuron. It is not trivial to perform EM and identify the AIY ectopic site in the mutant animals. In the revision, we examined the relationship between RIA and the ectopic AIY presynaptic sites in *eat-4(OE)* and high temperature cultivated animals. We found that the AIY presynaptic sites extend beyond the RIA posterior end **(S5J and S5K Fig)**, suggesting some ectopic AIY presynaptic structures are either onto other neurons, or without postsynaptic targets. We are still use "ectopic presynapses" or "ectopic synapses" because some of them are onto RIA, and the rest are also potentially real synapses.

2) More background information should be included in the Introduction section about cima-1 and the nature and function of the ectopic cholinergic synapses in AIY. For example, are the postsynaptic partners of the ectopic presynapses in zone 1 known? Is it known if the synapses are merely being misplaced from zone 1 to zone 2 with no change in the connections between AIY and its normal postsynaptic partners, or are the postsynaptic partners altered? Are there any known functional consequences of these ectopic presynapses or changes in "synaptic subcellular specificity"?

Answer:

The reviewer is asking the following three questions: (1) what is the postsynaptic partner of the AIY ectopic synapses; (2) Is the ectopic synapse in zone 1 from the misplacement of zone 2 synapse or newly established; (3) Do we see any synaptic subcellular specificity related function defect?

All are great questions. For (1), we don't have the answer. In wild-type animals, AIY form synaptic with RIA at zone 2 [1, 10, 11]. In *cima-1* mutants, AIY presynaptic structure partially overlaps with RIA, suggesting that some AIY ectopic synapses are onto RIA [1]. Our previous EM data showed that the ectopic AIY presynaptic sites are apposite to some unidentified neurons. However, we don't know whether those are the real postsynaptic neurons [1].

(2) Based on the morphology that the zone 2 region is smaller in *cima-1(wy84)*, *eat-4(OE)* or animals cultivated at 25°C, it is most likely that the zone 1 ectopic synaptic structure is originated from zone 2. We tried time-lapse to validate this. Unfortunately, we did not see the ectopic synaptic formation during the period of imaging. It is possible that the imaging condition suppresses the ectopic formation. We are optimizing the condition and are hoping that we could figure it out in the future.

(3) We have not yet tried hard to examine the behavior, but the overall locomotion appears

normal in *cima-1(wy84)*, *eat-4(OE)* or animals cultivated at 25°C.

3) Although this paper focuses on ectopic presynaptic markers in zone 1, data presented in several Figures suggest that the number or intensity of RAB-3 and/or SYD-1 puncta in zone 3 are also increased in cima-1 mutants (see Figures 1D and 1J). Do cima-1 mutants have an overall increase in presynapses throughout AIY in zones 1-3? In addition, overexpression of GLC-3 and GLC-4 appear to increase RAB-3 puncta in zone 3 and this effect is not dependent on eat-4 (Figures 4D-F). The authors should comment on these effects in zone 3.

Answer:

We did look the total synaptic intensity (zone 1-3) in *cima-1(wy84)* mutants previously, and found that the presynaptic intensity is reduced in the *cima-1(wy84)* mutants [1, 12]. To address the review's question, we quantified the synaptic intensity in zone 3 region, and found that the RAB-3 intensity in zone 3 is slightly reduced in the *cima-1(wy84)* mutants (**see the attached Fig. 1**). We now have replaced the images with more typical ones. Interestingly, the zone 3 GFP::RAB-3 intensity in *glc-3(OE);glc-4(OE)*animals is indeed increased, and this effect is *eat-4*-independent (**see the attached Fig. 1**). This result suggests that the presynaptic formation in zone 1 and zone 3 is differently regulated. We will further analysis this result in future paper since this MS is focusing on the zone 1/zone 2 region.

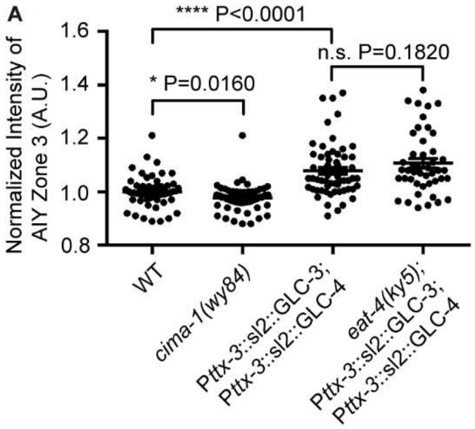

Fig. 1. (A) The normalized fluorescent intensity of AIY Zone3 for the indicated genotypes. Each data point represents a single independent animal from three biological replicates. Error bars are SEM. *P=0.0160, ****P<0.0001, n.s., not significant. Statistics were based on unpaired t test.

4) The synaptic connections illustrated in the EM images are not clear. (i) The electron dense presynaptic active zone structure in Figure 6A (Adult 3 bottom row) is not apparent. (ii) It is not clear that the electron dense structure marked by the arrowhead in Adult 2 (top row) is an active zone. The structure seems large and AIY does not appear to contact ASH. There is also another large dense structure nearby in the middle of the same neuron. (iii) The electron dense structure marked by the arrowhead in Adult 2 (bottom row) does not appear to contact AIY, but instead appears to make contact with another cell.

Answer:

Thank the reviewer for the comments. We agree that some EM images are not great. Since the EM data was recently published in bioRxiv [13], we revised the figure by showing one of the animals with 3-D ASH-AIY reconstruction and a series of sections to better illustrating the synaptic connection **(Fig 6A).**

5) The presence of GRASP signal in zone 3 in the nerve ring region of AIY is concerning. The EM reconstruction data shown in Figure 6A suggest that AIY and ASH make little if any contact in the nerve ring. This data suggests that expression of the GRASP constructs in ASH and AIY leads to abnormal contact between these neurons and perhaps disorganization of other processes in the nerve ring. These non-physiological contacts detract from the use of GRASP to illustrate contact between ASH and AIY in zone 1.

Answer:

Thank the reviewer for the comments.

Yes, we agree that the GRASP signal in zone 3 is concerning, therefore we took them out in the revised version. Thanks for the comments.

6) The paper would be strengthened if the authors could show that the effects of temperature on ectopic synapses are mediated by ASH. For example, does expression of eat-4 in ASH rescue the eat-4 suppression of high temperature induced ectopic synapses? Is the ASH process displaced posteriorly alongside AIY similar to what was observed in cima-1 mutants in Figure S7?

Answer:

Thank the reviewer for the comments.

In the revision, we performed ASH-specific *eat-4* rescue at high temperature and found that it indeed rescues. This data is now in **Fig 7R**.

We also examined the ASH processes at 25°C and found that they are displaced posteriorly alongside AIY **(S8C-S8C" Fig).**

Minor comments

1) There are many instances of incorrect grammar usage throughout the manuscript. These grammatical errors should be corrected.

Answer:

Thank the reviewer for the comments. We corrected those.

2) The rationale for investigating the effects of high cultivation temperature on ectopic synapses should be explained in more depth in the Introduction. For example, is there a connection between the role of AIY in thermotaxis and the effects of high cultivation temperature?

Answer:

Thank the reviewer for the comments.

One of the major reasons we examine the temperature effect is because AIY is in the thermotaxis circuit. In the revision, we added a paragraph **(third paragraph)** background in the introduction and explained this connection in the result section.

3) Overexpression of EAT-4 is not necessarily equivalent to “activating glutamate neurons” or “increase of glutamate release.” Even if more glutamate is loaded into presynaptic vesicles due to more copies of VGLUT/synaptic vesicle, this will not lead to activation of glutamate neurons. Is there evidence in the literature that overexpression of VGLUT is sufficient to increase glutamate release? If not, the authors should provide evidence that eat-4(OE) increases glutamate release or tone down their statements equating eat-4(OE) to “activating glutamate neurons or “increase of glutamate release” throughout the manuscript.

Answer:

Thank the reviewer for the comments.

Yes, we agree that EAT-4 overexpression is not equivalent to the “activating glutamate neurons”. We changed the wording in the revision.

Overexpressing VGLUT has been shown to increase the glutamate release in many animal models including *Drosophila*, mouse and rat [6, 8, 9]. We cited them in the revised version. To support test this possibility in *C. elegans*, we examined the spontaneous AIY activity by AIY-specific GCamP6s. Interestingly, we observed spontaneous Ca2+ oscillation in AIY. Notably, the frequency of Ca2+ oscillation in AIY is significantly reduced in ASH-specific *eat-4(OE)* worms **(Fig 4I-4K, S1 Video)**. Those data suggest that ASH-specific *eat-4(OE)* affects the AIY activity by over releasing glutamate.

4) In Figure 1D, zone 2 appears much smaller in cima-1 mutants. Are the presynaptic clusters spreading along the process into zone 1? If so, this information could inform the mechanism of how the ectopic synapses are forming.

Answer:

This is a great question. It is most likely that the zone 1 presynaptic structure is spreading from zone 2. However, we don’t have a solid data to support this hypothesis. We have performed a time lapse, but failed to capture the ectopic formation process. We are trying to optimize the condition so that we will be able to this question in near future.

5) Figure 5 shows that GLC-3 and GLC-4 colocalize with Rab-3, which is presumably on acetylcholine-containing synaptic vesicles. Are these receptors localized at presynapses in adults? And if so, the authors should comment on a physiological role for GLC-3 and GLC-4 at excitatory presynapses in the mature brain after synapses have formed. Are there examples from other systems where inhibitory receptors such as GABA receptors are localized at excitatory presynapses?

Answer:

Thank the reviewer for the comments.

Yes, the images were taken at adult stages.

Although GLC-3 and GLC-4 appear localized to presynaptic sites, they could be localized to the postsynaptic sites or both pre- and postsynaptic sites since those sites are aligned closely in the same region in AIY neurons (also see the response to Reviewer #2 question 4). This is now discussed in the discussion **(lines 482-491)**.

We don't know the physiological role of those presynaptic GLC-3/GLC-4. One possibility is that the presynaptic GLC-3/GLC-4 receptors regulate the local presynaptic excitability. As a matter of fact, GABA receptors has been shown localized to the glutamate presynaptic terminus in mammalian brain[14-17], which plays important roles in regulating neurotransmitter release.

6) The authors should tone down their conclusion that GLC-3 and GLC-4 act locally in zone 1 to regulate presynaptic assembly (line 286) as GLC-3 and GLC-4 could also act in zone 3 in the nerve ring where they are also localized.

Answer:

Thank the reviewer for the comments.

Although we don't have direct evidence to support GLC-3/GLC-4 acting locally to promote the ectopic synaptic assembly, we are pretty confident because of two lines of evidence. First, GLC-3/GLC-4 localized to or very close to the presynaptic sites, suggesting that GLC-3/GLC-4 most likely acts locally to regulate presynaptic assembly; secondly, glutamate from ASH, which form synapses onto the anterior border of zone 1 [13], but not other AIY presynaptic partners such as AFD that synapse onto the zone 3 region where is far away from zone 1 [10, 13, 18], promotes the ectopic presynaptic assembly in the zone 1 region. The GLC-3 and GLC-4 at the zone 3 region may promote the presynaptic assembly there locally **(see data for address question reviewer #3 question #3**), which supports the model that they act locally to promote the presynaptic assembly. Since we don't have direct evidence, we toned down the conclusion with "probably" **(line 345)**.

7) Zone 1 should be marked on the GRASP images in Figure 6. For example, it is not clear if the zone 1 region of AIY is visible in Figure 6E.

Answer:

Thank the reviewer for the comments.

In the revision, we deleted the GRASP images.

8) The term “N = number of animals, n = number of times scored” shown on the bar graphs should be clarified. Does this mean the same animal was scored several times and included in the data set or that N animals were scored over 6 different imaging sessions? Have all data underlying the figures and results presented in the manuscript been provided? Large-scale datasets should be made available via a public repository as described in the PLOS Genetics data availability policy, and numerical data that underlies graphs or summary statistics should be provided in spreadsheet form as supporting information.

Answer:

Thank the reviewer for the comments.

The term “n or n1” is number of biological replicates. “N” is the total number of assayed independent individuals. We made it clear in the revision. The original data set is provided in the **S3 Excel**.

References

1. Shao Z, Watanabe S, Christensen R, Jorgensen EM, Colon-Ramos DA. Synapse location during growth depends on glia location. Cell. 2013;154(2):337-350.

2. Thompson O, Edgley M, Strasbourger P, Flibotte S, Ewing B, Adair R, et al. The million mutation project: a new approach to genetics in Caenorhabditis elegans. Genome Res. 2013;23(10):1749-1762.

3. Lee RY, Sawin ER, Chalfie M, Horvitz HR, Avery L. EAT-4, a homolog of a mammalian sodium-dependent inorganic phosphate cotransporter, is necessary for glutamatergic neurotransmission in caenorhabditis elegans. J Neurosci. 1999;19(1):159-167.

4. Serrano-Saiz E, Poole RJ, Felton T, Zhang F, De La Cruz ED, Hobert O. Modular control of glutamatergic neuronal identity in C. elegans by distinct homeodomain proteins. Cell. 2013;155(3):659-673.

5. Lee D, Jung S, Ryu J, Ahnn J, Ha I. Human vesicular glutamate transporters functionally complement EAT-4 in C. elegans. Molecules and cells. 2008;25(1):50-54.

6. Daniels RW, Collins CA, Gelfand MV, Dant J, Brooks ES, Krantz DE, et al. Increased expression of the Drosophila vesicular glutamate transporter leads to excess glutamate release and a compensatory decrease in quantal content. J Neurosci. 2004;24(46):10466-10474.

7. Daniels RW, Miller BR, DiAntonio A. Increased vesicular glutamate transporter expression

causes excitotoxic neurodegeneration. Neurobiol Dis. 2011;41(2):415-420.

8. Wilson NR, Kang J, Hueske EV, Leung T, Varoqui H, Murnick JG, et al. Presynaptic regulation of quantal size by the vesicular glutamate transporter VGLUT1. J Neurosci. 2005;25(26):6221-6234.

9. Wojcik SM, Rhee JS, Herzog E, Sigler A, Jahn R, Takamori S, et al. An essential role for vesicular glutamate transporter 1 (VGLUT1) in postnatal development and control of quantal size. Proc Natl Acad Sci U S A. 2004;101(18):7158-7163.

10. White JG, Southgate E, Thomson JN, Brenner S. The structure of the nervous system of the nematode Caenorhabditis elegans. Philosophical transactions of the Royal Society of London Series B, Biological sciences. 1986;314(1165):1-340.

11. Colón-Ramos DA, Margeta MA, Kang S. Glia promote local synaptogenesis through UNC-6 (netrin) signaling in C. elegans. Science. 2007;318(5847):103-106.

12. Dong X, Jin S, Shao Z. Glia Promote Synaptogenesis through an IQGAP PES-7 in C. elegans. Cell Rep. 2020;30(8):2614-2626.e2612.

13. Witvliet D, Mulcahy B, Mitchell JK, Meirovitch Y, Berger DR, Wu Y, et al. Connectomes across development reveal principles of brain maturation in C. elegans. 2020:2020.2004.2030.066209.

14. Isaacson JS, Solís JM, Nicoll RA. Local and diffuse synaptic actions of GABA in the hippocampus. Neuron. 1993;10(2):165-175.

15. Isaacson JS. Spillover in the spotlight. Curr Biol. 2000;10(13):R475-477.

16. Manz KM, Baxley AG, Zurawski Z, Hamm HE, Grueter BA. Heterosynaptic GABA(B) Receptor Function within Feedforward Microcircuits Gates Glutamatergic Transmission in the Nucleus Accumbens Core. J Neurosci. 2019;39(47):9277-9293.

17. Sanchez-Vives MV, Barbero-Castillo A, Perez-Zabalza M, Reig R. GABA(B) receptors: modulation of thalamocortical dynamics and synaptic plasticity. Neuroscience. 2020.

18. Feinberg EH, Vanhoven MK, Bendesky A, Wang G, Fetter RD, Shen K, et al. GFP Reconstitution Across Synaptic Partners (GRASP) defines cell contacts and synapses in living nervous systems. Neuron. 2008;57(3):353-363.
