# Supplementary material for: Temperature regulates synaptic subcellular specificity mediated by inhibitory glutamate signaling
Source: PLoS Genet. 2021 Jan 11;17(1):e1009295. doi: 10.1371/journal.pgen.1009295 (PMC7822552; doi:10.1371/journal.pgen.1009295)
Supplement: Attachment — Submitted filename: response to reviewers.pdf [file pgen.1009295.s017.pdf]

Zhiyong Shao
Professor of Institutes of Brain Science Fudan University
138 Yixueyuan Rd, Mingdao Bldg 1016, Shanghai
Shanghai, 200032, China
Phone: +86-21-54237662
Email: shaozy@fudan.edu.cn

Point-by-point response to the reviewers' comments

Reviewer #1: The author's have satisfied my concerns with their thorough response. I approve of this manuscript for publication.

Response:
Thank the reviewer so much for the nice and positive comments.

Reviewer #2: They have addressed all my questions regarding experiments. It is a very interesting study, but the way the authors wrote the manuscript makes it difficult to follow. I will suggest a minor revision with a some corrections and clarifications on writing before acceptance for publication.

Response:
Thank the reviewer so much for the positive comments. We agree that our writing need to further improve. We carefully corrected all language errors we can find and rewrote some part in a way that could be easier to understand. The corrections include but not limited the following:

1) Line 25, we changed "…how **it affects** synaptic subcellular specificity…" into "…how **they affect** synaptic subcellular specificity…";
2) Line 49-50, we changed "synaptic targeting not only **chooses specific partner cells, but also specific subcellular compartments**" into "Synaptic targeting **happens not only at the cellular, but also at the subcellular level**";
3) Line 64, the "genetically encoded" in "…regulated by activity-independent **genetically encoded** mechanisms" has been deleted;
4) Line 73, "…and **transmit to the AIY** through glutamatergic synapses" has been changed into "…and **transmit it to the AIY interneurons** through glutamatergic synapses";
5) Line 86, "additional signals, most likely from the nervous system, **is** involved" has been changed into "additional signals, most likely from the nervous system, **are** involved";
6) Line 106, "The CIMA-1 in epidermal cells and the MIG-17 in **the muscle**…" has been changed into "The CIMA-1 in epidermal cells and the MIG-17 in **muscles**…";

7) We have changed all finding results into past tense;
8) Line 118, “Next, we want to **ask**…” has been changed into “Next, we **asked**…”;
9) Line 168-169, we added “**the**” in the front of “ASH” and “AIY”;
10) Line 170, “the” in the “To understand where **the** *eat-4* acts to regulate…” has been deleted;
11) Line 235, “Consistently, we found that the length and the ratio of ventral synaptic length are…” was changed into “Consistently, we found that the **ventral synaptic** length and the ratio of ventral to **total synaptic length were**…”;
12) Line 310, “Those data **revealed**…” has been changed into “Those data **reveal**…”;
13) Line 314, “we **speculate** that they **induce** the ectopic synapses through inhibiting AIY activity” has been changed into “we **speculated** that they **induced** the ectopic synapses through inhibiting AIY activity”;
14) Line 327, “the” in the “To further understand **the** how GLC-3 and GLC-4” has been deleted;
15) Line 450, the typo “GCL -3” in “…locates the **GCL-3**/GLC-4…” has been corrected;
16) Line 474, “Pentameric ligand-gated ion **channel family GLC-3 and GLC-4**” has been changed into “Pentameric ligand-gated ion **channels regulate synaptic specificity**”;
17) Line 508, “**Synaptic temporal and** spatial specificity” has been changed into “**Temporal regulation of** spatial specificity”;
18) Line 512, “we found that the amount of glutamate **mainly** released from ASH is **important** for the synaptic assembly in zone 1 region.” has been changed into “we found that the amount of glutamate released from ASH is **critical** for the synaptic assembly in zone 1 region”;
19) Line 516-521, “**In *eat-4(OE)* animals,** the ectopic synapses appear since newly hatched larval L1 stage. **The temporal difference of the ectopic synapse emergence between *cima-1(wy84)* and *eat-4(OE)* indicates that they may use** different molecular mechanisms.” has been changed into “The ectopic synapses appear since newly hatched larval L1 stage **in *eat-4(OE)* animals and at the adult stage in cima-1 mutants [55]. Additionally, the VCSC glia contribute more to the synaptic defect of *cima-1(wy84)* than that of *eat-4(OE)*. Those differences indicate that *cima-1(wy84)* and *eat-4(OE)* may regulate the synaptic subcellular specificity through** different molecular mechanisms.”.
20) Line 1194, “…and **wild-type animals expressed the gain of function UNC-103(A334T) in AIY**.” has been changed into “…and **AIY-specific *unc-103(gf)[UNC-103(A334T)]* animals**”;
21) Line 1251, “**wild-type animals do not have synapses in the AIY zone 1(white dashed box),** indicated with…” has been changed into “**the AIY presynaptic distribution is normal, as** indicated with…”;

22) Line 1253, “the ectopic synapses **show up**…” has been changed into “ the ectopic synapses **emerge in the zone 1 region**…”;
23) We have changed all “Statistics **were** based on unpaired t test.” into “Statistics **are** based on unpaired t test.”;
24) We have changed all “Quantification of the percentage of animals with the **ectopic AIY synaptic marker GFP::RAB-3**. ” into “Quantification of the percentage of animals with the **ectopic AIY synaptic marker GFP::RAB-3 in the zone 1 region**.”;
25) We have changed all “…are not required for synaptic subcellular specificity **at normal cultivation conditions**.” into “are not required for synaptic subcellular specificity **per se**.”;
26) Line 1339-1341, “…transcriptional reporter (P*glc-3*::GFP)**. P*glc-3*::GFP expression begins from embryo stage (C’). (C) is the corresponding bright field micrograph.**” has been changed into “…transcriptional reporter (*Pglc-3*::GFP) **at the embryonic stage (C’) and the corresponding bright field micrograph (C).”;**
27) Line 1342-1345, “…transcriptional reporter (P*glc-4*::GFP)**. P*glc-4*::GFP expression begins from embryo stage (D’). (D) is the corresponding bright field micrograph.**” has been changed into “…transcriptional reporter (*Pglc-4*::GFP) **at the embryonic stage (D’) and the corresponding bright field micrograph (D).”**.

Additionally, we modified a little in the abstract, author summary, introduction, results and discussion so that it is easier to read. Those include:
1) In the abstract, we replaced “… we found that **specific glutamatergic neurotransmission is involved in synaptic subcellular specificity. And high cultivation temperature robustly induces**…” with “we found that **high cultivation temperature robustly induces defects in synaptic subcellular specificity through glutamatergic neurotransmission**.”;
2) In the author summary, we deleted “**Overexpressing either *eat-4* or *glc-3/glc-4* results in the ectopic synaptic formation mimicking high temperature cultivation. Collectively**”;
3) We added a couple of examples in the first paragraph in the introduction to explain the synaptic subcellular specificity, and one transition sentence between the genetic factors and the environmental factors. We also deleted **“The ADAMTS protease MIG-17 in the body wall muscle is required for the VCSC displacement [55]. The displaced glia endfeet then contact the AIY asynaptic region ectopically and promote synaptic assembly at the contact sites through the neuronal expressed IQGAP/PES-7 and CDC-42 [56]**” to make it reads more smoothly.
4) We rewrote the *eat-4(OE)* (**Line 199-202**), *glc-3/glc-4* rescue (**Line 303-308**) results so that it reads better. We also changed the way transiting from genetic data to temperature assays (**Line 358-360**).

5) In the discussion, we deleted "**In previous study, we showed that CIMA-1 is required for synaptic subcellular specificity during postembryonic developmental stages because in the *cima-1(wy84)* loss-of-function mutants, the synaptic distribution is normal at larval stages, and becomes defects until the adult stage [55]. "** (Line 508-509), and "**To summarize, we uncovered that high temperature mainly during embryogenesis disrupts the synaptic subcellular specificity, which is mediated by glutamate from presynaptic neurons and the glutamate-gated chloride channels. Given that the involved environmental factor is common and molecules are highly conserved in many organisms, we speculate that this finding could provide novel insights into the high temperature induced neurological disorders**." (Line 548) to make it reads more smoothly and concisely.

Reviewer #3: The authors have added a significant number of informative experiments in the revised version of the manuscript including cell ablation of ASH, analysis of other stresses and measurement of GCaMP in AIY and VGLUT-pHluorin in ASH. All my concerns have been addressed by the authors.

Response:
Thank the reviewer so much for the nice and positive comments.
